# *HFE H63D* Polymorphism as a Modifier of the Effect of Cumulative Lead Exposure on Pulse Pressure: The Normative Aging Study

**DOI:** 10.1289/ehp.1002251

**Published:** 2010-05-14

**Authors:** Aimin Zhang, Sung Kyun Park, Robert O. Wright, Marc G. Weisskopf, Bhramar Mukherjee, Huiling Nie, David Sparrow, Howard Hu

**Affiliations:** 1 Department of Environmental Health Sciences, University of Michigan School of Public Health, Ann Arbor, Michigan, USA; 2 Department of Environmental Health, Harvard School of Public Health, Boston, Massachusetts, USA; 3 Channing Laboratory, Department of Medicine, Brigham and Women’s Hospital, Harvard Medical School, Boston, Massachusetts, USA; 4 Department of Emergency Medicine, Children’s Hospital, Harvard Medical School, Boston, Massachusetts, USA; 5 Department of Biostatistics, University of Michigan School of Public Health, Ann Arbor, Michigan, USA; 6 School of Health Sciences, Purdue University, West Lafayette, Indiana, USA; 7 Veterans Affairs Boston Healthcare System, Boston, Massachusetts, USA; 8 Boston University Schools of Public Health and Medicine, Boston, Massachusetts, USA

**Keywords:** gene–environment interaction, hemochromatosis gene, H63D mutation, lead exposure, pulse pressure

## Abstract

**Background:**

Cumulative lead exposure is associated with a widened pulse pressure (PP; the difference between systolic and diastolic blood pressure), a marker of arterial stiffness and a predictor of cardiovascular disease. Polymorphisms in the hemochromatosis gene (*HFE*) have been shown to modify the impact of cumulative lead exposure on measures of adult cognition and cardiac function.

**Objectives:**

We examined whether the *HFE* mutations modify the impact of lead on PP in community-dwelling older men.

**Methods:**

We examined 619 participants with a total of 1,148 observations of PP from a substudy of bone lead levels (a measure of cumulative exposure, measured by *in vivo* K-shell X-ray fluorescence) and health in the Normative Aging Study between 1991 and 2001. Linear mixed-effects regression models with random intercepts were constructed.

**Results:**

Of the 619 subjects, 138 and 72 carried the *HFE H63D* and *C282Y* variants, respectively. After adjusting for age; education; alcohol intake; smoking; daily intakes of calcium, sodium, and potassium; total calories; family history of hypertension; diabetes; height; heart rate; high-density lipoprotein (HDL); total cholesterol:HDL ratio; and waist circumference, baseline bone lead levels were associated with steeper increases in PP in men with at least one *H63D* allele (*p*-interaction = 0.03 for tibia and 0.02 for patella) compared with men with only the wild types or C282Y variant.

**Conclusions:**

The HFE *H63D* polymorphism, but not the *C282Y* mutation, appears to enhance susceptibility to the deleterious impact of cumulative lead on PP, possibly via prooxidative or pro-inflammatory mechanisms.

Pulse pressure (PP), the pulsatile component of blood pressure, is a surrogate marker of arterial stiffness and compliance. With increasing age, arteries stiffen because of loss of elasticity, calcification, and atherosclerosis in the vessel wall (Zieman et al. 2005). Increased PP has been shown in a number of studies to be a strong predictor of cardiovascular diseases, even after other risk factors were controlled for (Dart and Kingwell 2001). The mechanisms responsible for PP progress remain unclear. Whole genome and candidate gene analyses for PP have shown a significant genetic influence but have failed to identify specific allele determinants, probably because of the multifactorial etiology of arterial stiffness (Bielinski et al. 2005; Lacolley et al. 2009).

Although the relationship between lead exposure and increased blood pressure has been well established (Hu et al. 1996; Navas-Acien et al. 2007), the impact of lead on PP has been studied only to a limited extent. Lead can accumulate in the body after exposure, and the aorta is the second most lead-laden organ next to the skeleton in humans (Barry and Mossman 1970; Schroeder and Tipton 1968). As a prooxidant metal, lead has been implicated in atherosclerosis, peripheral vascular diseases, and other adverse cardiovascular end points (Navas-Acien et al. 2004, 2007). In our previous report (Perlstein et al. 2007), cumulative community-level lead exposure, as measured by *in vivo* measurements of lead in bone, was significantly associated with increased PP. The extent to which lead accumulation contributes to promoting vascular impedance is not clearly established.

Polymorphisms in the hemochromatosis gene (*HFE*) are well known to promote lifelong excessive iron absorption and accumulation and the risk of developing late-onset hereditary hemochromatosis, a common autosomal recessive, multisystemic disease. Two major mutations are known in the *HFE* gene: *C282Y* and *H63D* mutations. The protein encoded for by *HFE* gene binds to the transferrin receptor and reduces its affinity for iron-loaded transferrin and the subsequent cellular uptake of iron (Feder et al. 1998). The *H63D*-mutated protein (histidine-63-aspartate) appears to form stable complexes with the transferrin receptor and lowers its binding affinity for transferrin that leads to relatively small increments of cellular iron content, whereas *C282Y*-mutated protein (cystein-282-tyrosine) is expressed with unfolded/misfolded configuration and aggregates and remains in the endoplasmic reticulum (Gray et al. 2009). Homozygosity for *C282Y* variant is the main susceptibility genotype and is highly prevalent (50–100%) in hereditary hemochromatosis. Heterozygotes for *C282Y*, the homozygosity for variant *H63D,* and the compound heterozygotes of *H63D* and *C282Y* also succumb to hemochromatosis with a lower penetrance (Burke et al. 2000).

*HFE* mutations are also associated with altered lead biomarker concentrations (Wright et al. 2004) and modification of the impact of cumulative lead exposure on cognition (Wang et al. 2007), QT prolongation (Park et al. 2009), and air particle-related metal transport (Park et al. 2006) in elderly men, suggesting that protein encoded for by the *HFE* gene may modulate lead toxicity as well as iron uptake. These findings prompted us to hypothesize that the *HFE* gene mutation would modify the effects of lead on PP. Furthermore, because *H63D* and *C282Y* are known to have different penetrance rates (Burke et al. 2000), there may be a primacy of one *HFE* variant allele over the other in modifying lead toxicity. To address these issues, the aims of this study were to evaluate potential interactions between *HFE* genotypes and bone lead and to reconcile the difference of allele effects on PP in a subgroup of community-dwelling older men (those who are healthy, have no occupational or extra environmental risks from exposure to toxicants, and generally represent the population of the United States) from the Normative Aging Study (NAS).

## Materials and Methods

### Study population

The NAS is an ongoing longitudinal study of aging initiated by the U.S. Veterans Administration in 1963, when 2,280 healthy men, mostly white and 21–80 years of age, from the Greater Boston, Massachusetts, area were enrolled. All participants in the study had no history of heart disease, hypertension, diabetes, cancer, peptic ulcer, gout, recurrent asthma, bronchitis, or sinusitis. Those with either a systolic blood pressure > 140 mmHg or a diastolic blood pressure > 90 mmHg were disqualified for enrollment. Between August 1991 and December 1995, 744 NAS participants who gave their informed consent were invited to undergo bone lead measurements. Of those subjects in the lead study, 632 were successfully genotyped for *HFE* gene. To evaluate effect modification by allele-specific *HFE* variances, men heterozygous for both *C282Y* and *H63D* polymorphisms (known as compound heterozygote; *n* = 13) were excluded; thus, 619 participants were finally included in the study. Complete medical and family histories, physical examinations, and laboratory tests closest to the time point of bone lead measurements were defined as baseline measurements. Every 3–5 years, the participants were scheduled for follow-up examinations. Between August 1991 and December 2001, there were 529 follow-up examinations among those 619 participants; therefore, a total of 1,148 observations were used in the analysis (246, 217, and 156 subjects have one, two, and three observations, respectively). The study protocol was approved by the institutional review board at each study site.

### Blood pressure measurements

During each clinical visit, the participants came to the study center in the morning after abstaining from smoking and drinking for at least 12 hr. Immediately after the history review, while the subject remained seated, systolic blood pressure and fifth-phase diastolic blood pressure were measured to the nearest 2 mmHg, first in the left arm and then in the right arm, with a standard mercury sphygmomanometer with a 14-cm cuff. For this study, the means of the left and right arm measurements were used as systolic and diastolic blood pressure for each participant, and PP was calculated as the systolic minus the diastolic blood pressure.

### Laboratory measurements

Bone lead levels were measured closest in time to the first visit using a K-shell X-ray fluorescence instrument (ABIOMED, Danvers, MA) at two sites: the midtibial shaft and the patella, which represent the cortical and trabecular bone compartments (Hu et al. 1996; Kim et al. 1995). Multiplex polymerase chain reaction assays were designed with Sequenom SpectroDESIGNER software (Sequenom, Inc, San Diego, CA) by inputting sequence containing the single-nucleotide polymorphism (SNP) site and 100 bp of flanking sequence on either side of the SNP, as previously described (Park et al. 2006; Wright et al. 2004). For further measurement details, see Supplemental Material (doi:10.1289/ehp.1002251).

### Covariates

Covariates were chosen based on biology, potential mediating effects, and prior studies (Hu et al. 1996; Perlstein et al. 2007). Information about age, race, and ethnicity, as well as medical and family history were obtained from the interview. Information about heart rate, body mass index (BMI) waist circumference, physical activities, and alcohol use were obtained from the physical examination and examination-associated questionnaire, respectively. The identity and purpose of medications prescribed by the subject’s physician were confirmed. Dietary intake was assessed using a standardized food frequency questionnaire. In the present study, the nutrients examined, such as potassium, sodium, and calcium, were adjusted for total energy intake. Information about education and cigarette smoking was also obtained by questionnaire.

### Statistical analysis

Statistical analysis was carried out using SAS (version 9.1; SAS Institute Inc., Cary, NC) and R version 2.7.2. (R Foundation for Statistical Computing, Vienna, Austria), and the primary outcome measure was PP. All probability measures corresponding to statistical significance are two tailed (α = 0.05). We used the generalized extreme studentized deviation many-outlier method (Rosner 1983) to remove extreme outliers among the continuous independent variables with a normal distribution, as in previous analyses. Given the high penetrance of two *HFE* variant alleles over single *HFE* variant alleles, it is possible that the magnitudes of effect modification among *H63D* homozygotes or *C282Y* homozygotes is larger than that among *H63D* heterozygotes or *C282Y* heterozygotes. However, because there were small sample sizes in *HFE* homozygote genotype, we were unable to test alleles dose responses. Instead, we classified the *HFE* genotypes into two different manners: binary [wild type (having only *HFE* wild-type alleles) and any *HFE* variant allele], and allele-specific [wild type, and mutually exclusive *C282Y* (having one or two *C282Y* variant allele) and *H63D* (having one or two *H63D* variant alleles)] in this study. Compound heterozygotes were excluded in all analyses. Allele and genotype frequencies and tests for Hardy–Weinberg equilibrium were performed. Because PP was measured repeatedly across time in the same individual, we fit a linear mixed-effects regression model with random intercepts using the PROC MIXED procedure in SAS to account for lack of independence of observations and the subject-specific intercept that reflects unexplained heterogeneity in the overall intercept (Singer and Willett 2003). To assess effect modification by the different *HFE* alleles, we included a term for the lead biomarker, indicator variables for *HFE* genotype classification, and cross-product terms between lead biomarker and *HFE* genotype, along with baseline age (years), age squared, time elapsed from the baseline (years), height (centimeters), education, alcohol intake (grams per day), smoking (pack-years), daily intakes of calcium, sodium, and potassium (milligrams), total calories (kilocalories per day), physical activity, diabetes, family history of hypertension, heart rate, high-density lipoprotein (HDL, milligrams per deciliter), total cholesterol-to-HDL ratio, and waist circumference (centimeters). We fit additional models that further adjusted for hypertension, ischemic heart disease, and diabetes mellitus as well as medications for hypertension, diabetes, and hyperlipidemia. We initially included an interaction between time and bone lead marker in the mixed-effects model to capture the longitudinal association between bone lead and PP over time. We excluded the interaction term because it was not significant, and it is difficult to test effect modification by HFE genotype.

The mixed model we used is described as follows:


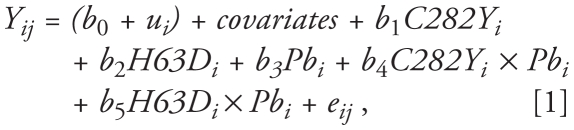


where *Y_ij_* is PP in subject *i* at visit *j*, *b*_0_ is the overall intercept, *u_i_* is a subject-specific intercept that reflects unexplained heterogeneity in the overall intercept, and *b*_1_,.., *b*_5_ are the slopes representing the estimated effects of *C282Y HFE* and *H63D HFE* compared with wild-type carriers, and the estimated effects of lead among wild type, *C282Y HFE,* and *H63D HFE*, respectively. To check whether the associations between PP and each lead marker were linear, we used the generalized additive mixed model (GAMM) with bone lead marker as penalized spline, which allows the relation between the exposure and response to be more flexible and thus allows for possible nonlinearity of the association. This was implemented using the R software.

## Results

Table 1 shows the genotype frequencies along with tibia and patella lead levels at baseline by *HFE* genotypes. Median concentrations of bone lead in our study population were 19 and 26 μg/g for tibia and patella, respectively. The distributions of both *H63D* and *C282Y* genotypes were in Hardy–Weinberg equilibrium (*H63D*: χ^2^ = 2.97, *p* = 0.09; *C282Y*: χ^2^ = 1.17, *p* = 0.36), which is consistent with the previously reported prevalence for *HFE* genotypes in the NAS cohort (Wright et al. 2004). Because of the small numbers in both *H63D* and *C282Y* homozygotes groups, our study population was further merged into two categories indicating the presence of any *H63D* or *C282Y* allele. As a result, 138 (21.8%) and 72 (11.4%) men had at least one *H63D* variant allele and *C282Y* variant allele, respectively. There were no differences in bone lead levels among *HFE* wild type, *H63D*, and *C282Y* variant carriers.

Table 2 presents baseline information of characteristics for the NAS men in our study sample (*n* = 619). Subjects with *HFE* variant had lower levels of HDL cholesterol and were more likely to have diabetes. *C282Y* variants had higher intake of calories and lower intake of calcium compared with *H63D* and wild-type subjects. Although systolic blood pressure and PP levels appeared higher in the *H63D* variant carriers, these measures were not significantly different from *C282Y* variant carriers or wild types. Compared with the men excluded (missing *HFE* genotype; *n* = 112), the men in our study sample were younger (66 vs. 70 years), had lower tibia and patella bone lead levels (19 vs. 22 μg/g and 26 vs. 32 μg/g for tibia and patella lead levels, respectively), and were less likely to have diabetes and use hypertension medications than those excluded (6.1% vs. 12.5% and 8.5% vs. 16.9% for diabetes prevalence and use of hypertension medications, respectively. [See Supplemental Material, Table 1 (doi:10.1289/ehp.1002251) for details.] As expected, there was an average of 2-mmHg increases in PP of the study participants during the follow-up period (mean follow-up years = 4.9 ± 5.8).

We examined the associations of *HFE* alleles with PP independent of lead exposures (data not shown). After controlling for the same potential confounders listed in “Statistical Analysis,” no *HFE* gene variants alone (either binary or allele-specific *HFE* variants) were significantly associated with PP. In the previous cross-sectional analysis (Perlstein et al. 2007), bone lead levels, not blood lead levels, were significantly associated with increased PP. In this expanded follow-up with a total of 1,148 observations, we did not find a significant interaction between time elapsed and tibia lead or patella lead in the determination of PP. Therefore, we removed the interaction term between time and bone lead from the model when we tested the interaction between bone lead and *HFE* genotype. Coefficients for any *HFE* variant effects on PP were not substantially changed after the addition of either tibia or patella lead to each of the PP models. In the multivariable regression models that included an interaction term between baseline bone lead levels and an indicator for having any *HFE* variant alleles, we found a significantly positive interaction for PP. Interestingly, in additional regression analyses including either *H63D* or *C282Y* separately, the observed interaction remained consistently significant only for *H63D*, not for *C282Y*. Higher tibia or patella lead was associated with a steeper increase in PP among the *H63D* participants compared with *C282Y* or wild-type men (*p* < 0.05) (Table 3). Among men with at least one *H63D* allele, interquartile range (IQR) increases of the lead levels in tibia (13 μg/g) and patella (19 μg/g) were associated with 3.3 mmHg [95% confidence interval (CI), 0.16–6.46 mmHg) and 2.95 mmHg (95% CI, 0–5.92 mmHg) increases in PP, respectively]. Although bone lead levels were also associated with PP elevation among *C282* variant and wild-type subjects, those associations were small in magnitude and not statistically significant.

**Figure 1:**
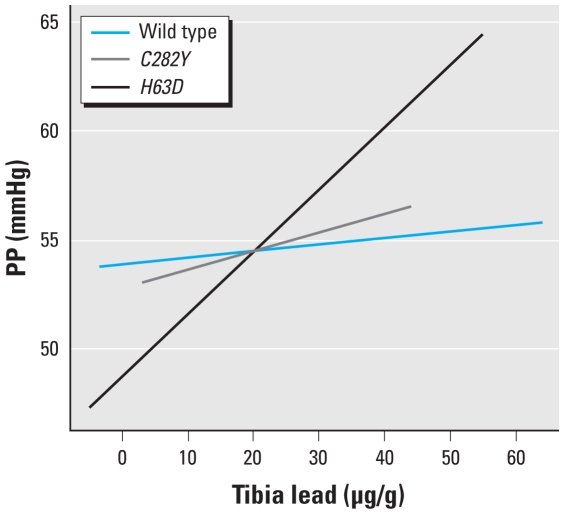
Adjusted association between tibia lead levels and change in PP by *HFE* genotypes in 619 community-exposed men with a total of 1,148 observations from the GAMM with interactions between HFE genotypes and tibia lead fit as penalized spline that allows the relation between the exposure and response to be more flexible and thus allows for possible nonlinearity of the association. The covariates adjusted for are the same as in Table 3.

In an adjusted plot of PP versus tibia lead stratified by the *HFE* genotype that was done using GAMM (Figure 1), we found that the estimated degrees of freedom for the penalized splines of the tibia lead to predict PP in all three *HFE* genotype groups were 1, which suggests that there was no evidence of a nonlinear shape to the dose–response relationship between tibia lead levels and PP, with the steepest slope in *H63D* subjects and a more modest slope in *C282Y* variants compared with the wild-type subjects. Similar modifying effects were noted in the association of patella lead with PP (data not shown).

Because blood pressure and cholesterol level in blood could influence arterial stiffness, one might be concerned that treatment for hypertension and hyperlipidemia could alter any effect modification by *HFE* variants. We examined this possibility by including a term for use of the related medications at the baseline in our study population in the multivariate models and also repeating the analyses after further excluding subjects who took the medications for those diseases. These models yielded similar results in the interaction of bone lead with *H63D* variant. Further adjustment for diagnosis of diabetes and ischemic heart disease at the baseline did not change the results (data not shown). The same was also true for analysis-restricted Caucasians (data not shown).

## Discussion

Although public health initiatives have been successful in decreasing environmental lead exposures in the United States and other industrialized nations, recent reports using data from the Third National Health and Nutrition Examination Survey (NHANES III) (Menke et al. 2006; Schober et al. 2006) as well as the NAS (Weisskopf et al. 2009) suggest that even low-level environmental lead exposure is associated with all-cause and cardiovascular mortality. Moreover, genetic variations render some people especially susceptible to lead’s toxicity (Onalaja and Claudio 2000), and identifying such heightened susceptibility is important not only for a better understanding of the etiologic relation between lead and adverse cardiovascular outcomes in older adulthood, but also for more accurately defining preventive and therapeutic measures. In the present longitudinal study of 619 older, community-dwelling men, we demonstrated a modifying influence of *HFE* genotype on the relation between cumulative lead burden and PP, after adjusting for potential confounders. We found that the linear relationship of PP with bone lead was particularly strong among *H63D* variant carriers compared with *C282Y* variant carriers and wild-type participants. That is, individuals carrying the *H63D* allele showed the strongest PP increases in relation to bone lead levels—even though they also had somewhat lower bone lead levels overall. As we have recently demonstrated and discussed in our lead and gene interaction models of cognition and electrocardiographic conduction (Park et al. 2009; Wang et al. 2007), the observed difference between the two *HFE* variants provides striking evidence of an allele-specific predisposition to lead toxicity and suggests that individuals with *H63D* variants may have a significantly higher risk for lead-increased PP. Given that a 10-mmHg wider PP increases the risk of major cardiovascular complications and mortality by nearly 20% (Blacher et al. 2000), the apparent lead effects, if causal, could account for approximate 6.6% risk of cardiovascular disease. Moreover, the findings from our present and previous studies underscore the importance of considering both genetic and environmental factors in the mechanisms involved in a multifactorial condition such as increased arterial stiffness, rather than the typical approach of evaluating one component or the other.

The progression of PP with advancing age is largely attributed to progressive central arterial stiffening as a consequence of altered vascular structure and function with disruption of elastin, increased collagen, calcification, and atherosclerosis. Lead accumulation in the human aorta (Barry and Mossman 1970; Schroeder and Tipton 1968) may contribute to accelerating arterial degenerative processes by reducing availability of nitric oxide and promoting oxidative damage and vascular inflammation (Vaziri and Sica 2004), purported mechanisms of vascular aging (Touyz 2004). It is possible that acceleration of PP may result from extra-osseous deposition of lead released from aged bone, and *HFE* genotypes affect PP elevation by modulating internal lead distribution between bone and a vulnerable arterial tree. However, the findings of a stronger association of PP with tibia lead than with patella lead (Perlstein et al. 2007) make this possibility somewhat less likely. The patella is composed mostly of trabecular bone, which has a higher reabsorption rate and is considered the major skeletal source of circulating lead (Hu 1998); thus, patella bone lead would have been expected to exert the greatest impact on PP if the mechanism of that impact required mobilization of bone lead stores. By contrast, the tibia is a cortical bone with a slow rate of bone turnover and a longer half-life with respect to lead levels. The stronger association of tibia lead with PP is consistent with bone lead as a proxy dosimeter for arterial lead accumulation and an effect of chronic lead exposure on vascular structure and function. Given the fact there was no significant difference in bone lead levels by *HFE* genotypes in the present study, toxicokinetic modifications seems unlikely to account for the apparent *H63D* effects, although its role in transporting lead into the blood vessel wall is unknown.

Both polymorphisms are common in the U.S. population (Burke et al. 2000; Wright et al. 2004), and the prevalence estimates for *HFE* variants are 5.4–17% for *C282Y* and 10–32% for *H63D*, respectively, which are similar to the prevalence rates in our study population. *C282Y* variants have been known to accumulate more iron in the body than H63 variants or wild-type subjects. Because the excess iron accumulation over physiological need (iron overload) has been implicated in atherosclerosis (Sullivan 1981) and arterial stiffness (Cheung et al. 2002), the acceleration of PP may be a central vascular outcome of synergistic interaction between lead and altered iron metabolism or endoplasmic reticulum stress in *HFE* variants. As such, one might have expected a greater PP elevation per unit increase in body lead burden among *C282Y* variants compared with *H63D* variants. However, the current data suggest quite the opposite, indicating that *H63D* variant augments the vascular toxicity of lead and appears to be independent of iron load pathway.

Findings from *in vitro* studies of human neuronal cells demonstrate an *HFE* gene pleiotropic effect: The *H63D* variant enhances, whereas *C282Y* decreases, oxidative stress and production of monocyte chemoattractant protein-1 (MCP-1) (Lee et al. 2007; Mitchell et al. 2009). More interestingly, *H63D* associations with MCP-1 were also observed in patients with hemochromatosis *in vivo* (Lawless et al. 2007). MCP-1 is a potent chemokine secreted by vascular or circulating cells and plays an important role in the regulation of leukocyte trafficking. It has been proposed that the pathogenesis of atherosclerosis is associated with an inflammatory response characterized in part by excessive recruitment of leukocytes from blood stream into the vessel wall (Braunersreuther et al. 2007). The relationships between *H63D* and MCP-1 and oxidative stress suggest another mechanism: that the modified association of *H63D* mutation with lead may be related to increased MCP-1–mediated inflammatory responses. Although the underlying mechanism(s) is not clear, the common prooxidant and proinflammatory tendencies shared by lead and the *H63D* genotype would clearly be consistent with biologic plausibility: the putative interaction between lead and *H63D* variant enhances vascular damage and inflammatory responses and, consequently, accelerates atherosclerosis and PP progress. Because we were not able to assess body redox status and MCP-1 and other inflammatory mediators in the blood, we cannot test this hypothesis in the statistical analysis.

It is noteworthy that two population studies, the Hemochromatosis and Iron Overload Screening and the Atherosclerosis Risk in Communities, reported that *C282Y* homozygosity was associated with lower total and low-density lipoprotein (LDL) cholesterol levels (Adams et al. 2009; Pankow et al. 2008). The failure of the *C282Y* variants to modify the relationship between lead and PP may be, in fact, the results of the perturbation of the body lipid metabolism in *C282Y* variants that may thus protect against arterial stiffening. The precipitation of LDL onto the arterial wall is the first key step to initiate vascular inflammatory responses and the development of fatty streaks and atherosclerotic plaques. Thus, lower LDL reduces arterial wall inflammation and should be associated with lower blood vessel stiffness. In our analysis, the LDL levels in *C282Y* and *H63D* variant carriers appeared to be lower than those in *HFE* wild types, but this was not statistically significant (Table 2). Thus, it is unlikely that this can explain the current finding.

Regardless of the actual mechanism, this study suggests a molecular link between accelerated PP and lead exposure and raises the possibility that *H63D* mutation is a previously unrecognized genetic risk factor for accelerated arterial aging. Because the progressive increase in arterial stiffness, marked by increased PP, is the common pathway of multiple risk factors that lead to similar cardiovascular diseases [e.g., left ventricular hypertrophy, myocardial infarction, and congestive heart failure (Zieman et al. 2005)], the modified associations between community exposure to lead and PP by common genetic variant, *H63D*, is compelling and has public health relevance. Since the discovery of the *HFE* gene more than a decade ago (Feder et al. 1998), its role in cardiovascular diseases has been studied intensively but has yielded conflicting results. Our study, in conjunction with the known high prevalence and low penetrance of the *HFE* gene, strongly suggests that discrepancies across studies may reflect that *HFE* polymorphisms may not independently predict such pathology, but may instead enhance the influence of environmental factors, such as lead, on the development of cardiovascular diseases in individuals who carry *HFE* mutations. Moreover, because both lead and *H63D* have been linked to Alzheimer’s disease (Berlin et al. 2004; Zawia et al. 2009), amyotrophic lateral sclerosis (Kamel et al. 2002; Sutedja et al. 2007), and male infertility (Benoff et al. 2000; Gunel-Ozcan et al. 2009), our work underscores the need for examination of the interaction of *H63D* mutation and lead exposure as a potential mechanism of other chronic diseases.

The strengths of this study include considerable follow-up that has enabled us to minimize the potential for biases that are often encountered in cross-sectional or case–control studies and to establish more clearly the temporal nature of the association. The NAS cohort was established about 35 years before the discovery of *HFE*; thus, participants had not been previously screened for mutations or selected based on knowledge of their genotype.

Our study has several limitations. Because the NAS study was conducted almost exclusively in white men, the conclusion from our study may be not applicable to women and nonwhite people. As in any study of genetic association, population substructure might produce the observed interaction; however, the present study population is very homogeneous, with 95% being of European descent. We cannot rule out residual or unmeasured time-dependent and time-independent confounding factors and interactions with other genetic polymorphisms or environmental factors. Other considerations include the limited number of *C282Y* or *H63D* homozygotes, possible *HFE* gene disequilibrium with other measured covariates and limited information on clinical diagnosis of hemochromatosis, and markers of inflammation (MCP-1) or iron metabolism (serum ferritin and transferrin saturation levels). The *HFE* gene is located on chromosome 6 (6p21.3) (Feder et al. 1998). We cannot exclude a possibility that the apparent effect modification may be caused by gene(s) in proximity to the *HFE* gene in chromosome 6 that influence or regulate PP or resulted from effects of less-common *HFE* variants, such as S65C (Pedersen and Milman 2009). Although possible, we think that those two possibilities are very unlikely. In either case, the impact should be seen in all subjects and not limited to *H63D* subjects as seen in our study. We did several sensitivity analyses by further controlling for diagnoses of hypertension and diabetes and treatments of hypertension and hyperlipidemia collected at baseline. Because of incomplete follow-up information on these variables in the data set that we examined in the present study, such sensitivity analyses may not completely rule out the possible confounding effect of diabetes, hypertension, or use of antihypertensive and lipid-lowering drugs.

In conclusion, our study of 619 older, community-dwelling men provides the first evidence that the *H63D* mutation significantly modifies the association between bone lead and PP. These observations support the notion of combined influences of genetic susceptibility and environmental lead exposure on the development of arterial stiffness.

## Figures and Tables

**Table 1:** Genotype frequencies along with bone lead levels [median (interquartile range)] by *HFE* genotype.

Genotype	No. (%)	Tibia lead (μg/g)	Patella lead (μg/g)
Wild type	409 (66.1)	18 (12–27)	26 (17–37)
*C282Y* heterozygotes	67 (10.8)	20 (14–26)	25 (17–37)
*C282Y* homozygotes	5 (0.8)	34 (16–39)	34 (22–58)
*H63D* heterozygotes	122 (19.7)	19 (14–27)	27 (19–37)
*H63D* homozygotes	16 (2.6)	17.5 (13.5–21.5)	26.5 (18.5–37)
*C282Y* variants^a^	72 (11.6)	20 (14–27)	25 (17–37)
*H63D* variants^b^	138 (22.3)	19 (14–26)	27 (19–37)
Any *HFE* variants^c^	210 (33.9)	19 (14–27)	26 (18–37)
All subjects	619 (100)	19 (13–27)	26 (18–37)

a Presence of any *C282Y* variant.

b Presence of any *H63D* variant.

c Presence of any *C282Y* or *H63D* variant.

**Table 2:** Characteristics of men at the baseline lead measurements by *HFE* genotype.

Characteristic	Wild type (*n* = 409)	*C282Y* (*n* = 72)	*H63D* (*n* = 138)	*p*-Value
Continuous variables (mean ± SD)				
Age	66.4 ± 7.0	66.9 ± 7.1	66.9 ± 6.8	0.78
BMI	27.8 ± 3.6	27.8 ± 3.6	27.6 ± 3.8	0.90
SBP (mmHg)	135.0 ± 17.1	134.5 ± 17.2	137.4 ± 18.9	0.51
DBP (mmHg)	81.5 ± 9.5	80.9 ± 9.2	81.9 ± 9.8	0.78
PP (mmHg)	53.5 ± 14.4	53.7 ± 14.4	55.4 ± 14.8	0.57
Heart rate (beats/min)	72.7 ± 9.7	72.1 ± 9.9	72.1 ± 11.7	0.66
Height (m)	1.73 ± 0.07	1.74 ± 0.06	1.73 ± 0.06	0.15
Fasting glucose (mg/dL)	107.6 ± 26.7	112.4 ± 35.1	110 ± 35.1	0.34
Hematocrit	43.8 ± 3.1	44.1 ± 3.4	43.7 ± 2.9	0.58
Hemoglobin (g/dL)	15.1 ± 1.2	15.4 ± 1.2	15.2 ± 1.1	0.29
LDL (mg/dL)	112.9 ± 37.1	111.9 ± 39.6	109.1 ± 39.7	0.73
HDL(mg/dL)	49.4 ± 13.5	46.8 ± 11.2	46.1 ± 10.9	0.03
Total/HDL ratio	4.93 ± 1.43	5.1 ± 1.58	5.2 ± 1.45	0.23
Alcohol (g/day)	14.2 ± 18.5	14.2 ± 18	11.9±17.3	0.37
Smoking (pack-years)	20.0 ± 24.0	24.6 ± 28.6	23.3 ± 26.8	0.20
Calories (kcal)	1,995 ± 594	2,106 ± 626	1,975 ± 718	0.02
Calcium (mg)	780 ± 353	905 ± 443	768 ± 358	0.02
Potassium (mg)	3,255 ± 992	3,510 ± 465	3,389 ± 1379	0.14
Sodium (mg)	3,757 ± 1,579	3,951 ± 1,816	3,773 ± 1,878	0.69

Categorical variable [*n* (%)]				
Education				0.16
Unfinished high school	43 (10.5)	15 (6.9)	9 (6.5)	
High school	238 (58.2)	52 (72.2)	87 (63.0)	
College and above	128 (31.3)	15 (20.8)	42 (30.4)	
Family history of HTN	157 (38.4)	30 (41.7)	51 (36.9)	0.80
Hypertension	164 (40.1)	29 (40.3)	62 (44.9)	0.61
Ischemic heart diseases	59 (14.4)	15 (20.8)	28 (20.3)	0.16
Diabetes	18 (4.4)	6 (8.33)	15 (10.8)	0.02
Hypertension treatment	34 (8.3)	8 (11.1)	12 (8.7)	0.74
Hyperlipidemia treatment	48 (11.7)	6 (8.3)	17 (12.3)	0.66
Diabetes treatment	13 (3.2)	5 (6.9)	10 (7.3)	0.08

Bone lead levels [median (IQR)]				
Tibia lead (μg/g)	18 (12–27)	20 (14–27)	19 (14–26)	0.31
Patella lead (μg/g)	26 (17–37)	25 (17–37)	27 (19–37)	0.94

Abbreviations: DBP, diastolic blood pressure; HTN, hypertension; IQR, interquartile range; LDL, low-density lipoprotein; SBP, systolic blood pressure.

**Table 3:** Adjusted parameter estimators in the difference of PP (mmHg) for an IQR increase in bone lead marker by *HFE* genotype.

Genotype	Subjects (*n*)	Total observations	Tibia lead IQR = 13 μg/g β (95% CI)	Patella lead IQR = 19 μg/g β (95% CI)
Wild types	409	755	0.38 (0–1.96)	0.26 (0–1.78)
*H63D*	138	256	3.30 (0.16–6.46)	2.95 (0–5.92)
*C282Y*	72	137	0.89 (0–5.24)	0.55 (0–1.66)
Any *HFE*^a^	210	393	2.90 (0.31–5.51)	2.83 (0.32–5.37)

All models were adjusted for baseline age; age squared; time elapsed from the baseline; height; education; alcohol intake; smoking; daily intakes of calcium, sodium, and potassium; calorie intake; physical activity; diabetes; family history of hypertension; heart rate; HDL; total cholesterol:HDL ratio; and waist circumference.

a Includes *H63D* and *C282Y* variants only.
